# Prefrontal and posterior parietal contributions to the perceptual awareness of touch

**DOI:** 10.1038/s41598-019-53637-w

**Published:** 2019-11-18

**Authors:** M. Rullmann, S. Preusser, B. Pleger

**Affiliations:** 1 Department of Neurology, Max Planck Institute for Human Cognitive and Brain Sciences, Stephanstr. 1a, 04103 Leipzig, Germany; 2 Department of Nuclear Medicine, University Hospital Leipzig, Liebigstr. 20, 04103 Leipzig, Germany; 3 Department of Neurology, BG University Hospital Bergmannsheil, Ruhr-University Bochum, Bürkle-de-la-Camp Plz. 1, 44789 Bochum, Germany; 4 Collaborative Research Centre 874 Integration and Representation of Sensory Processes, Ruhr-University Bochum, Universitätsstr. 150, 44801 Bochum, Germany

**Keywords:** Sensory processing, Brain

## Abstract

Which brain regions contribute to the perceptual awareness of touch remains largely unclear. We collected structural magnetic resonance imaging scans and neurological examination reports of 70 patients with brain injuries or stroke in S1 extending into adjacent parietal, temporal or pre-/frontal regions. We applied voxel-based lesion-symptom mapping to identify brain areas that overlap with an impaired touch perception (i.e., hypoesthesia). As expected, patients with hypoesthesia (n = 43) presented lesions in all Brodmann areas in S1 on postcentral gyrus (BA 1, 2, 3a, 3b). At the anterior border to BA 3b, we additionally identified motor area BA 4p in association with hypoesthesia, as well as further ventrally the ventral premotor cortex (BA 6, BA 44), assumed to be involved in whole-body perception. At the posterior border to S1, we found hypoesthesia associated effects in attention-related areas such as the inferior parietal lobe and intraparietal sulcus. Downstream to S1, we replicated previously reported lesion-hypoesthesia associations in the parietal operculum and insular cortex (i.e., ventral pathway of somatosensory processing). The present findings extend this pathway from S1 to the insular cortex by prefrontal and posterior parietal areas involved in multisensory integration and attention processes.

## Introduction

The primary somatosensory cortex (S1) in monkeys can be divided into four Brodmann areas: (BA) 1, 2, 3a, and 3b. Each BA consists of a somatotopically organized map that subserves distinct somatosensory functions^1–3^. BA 3b^4^ and BA 1^5^ receive their main input from cutaneous receptors, while distinct subdomains respond to different sensory stimuli, such as vibration, flutter, or pressure^6^. Also, BA 3a receives cutaneous inputs, but its activation is predominately driven by muscle receptors^4,7^. Nevertheless, damage to either BA 3b or BA 3a severely impairs somatosensory perception^8^. Lesions within BA 2 also cause impaired perception, but rather of kinaesthetic inputs^8^ since its afferents mainly originate in joints and muscles^9^. Human lesion studies are in general agreement with these observations, although brain lesions in humans, due to stroke or trauma, do not affect single BAs, so that their distinct functional implementation remains less clear. Like S1 proper in non-human primates, BA 3b in humans seems to receive the main thalamic output^10^, and lesions comprising BA 3b or adjacent BAs on S1 can cause an impaired perception of shape and space^11–13^.

From S1, somatosensory information is assumed to be projected to the parietal operculum (OP, secondary somatosensory cortex, S2), before terminating in the insular cortex^3^. In a recent virtual lesion-symptom mapping (VLSM) study, we provided causal proof for the existence of this pathway^14^. Attenuated perception of touch was associated with lesions comprising the contralateral parietal operculum (OP), predominantly the more frontally located subdivisions OP 4 and 3, together with the insula, the putamen, and white matter projections to the prefrontal cortex. These findings were subsequently replicated in stroke patients with the same MRI-based VLSM methods^15^.

In the present VLSM study, we addressed the question which brain regions upstream to the parietal operculum, i.e. S2, underpin the perceptual awareness of touch. In line with previous research^11–13^, we hypothesized to identify the four sub-divisions of S1, i.e., BA 1, BA 2, and BA 3a/b, as well as the inferior parietal lobe (i.e., IPL) together with the intraparietal sulcus (i.e., IPS) involved in calibrating somatosensory attention^16,17^. We did not assume to identify any associations to regions in the motor cortex, such as BA4a. Downstream to S1, we assumed to replicate previous VLSM findings including the parietal operculum, OP 1–4, together with the insular cortex^3,14,15^.

## Results

MRI scans and examination reports were collected by the Clinic of Cognitive Neurology, University Clinic Leipzig, and Max Planck Institute (MPI) for Human Cognitive and Brain Sciences in Leipzig, Germany. According to the inclusion and exclusion criteria (see “*Patients*” in Methods, Supplementary Table 1), we identified 35 patients (22 men, 13 women; average age = 46.82 ± 12.12 years) with brain lesions in the left hemisphere and 35 patients (28men, 7 women; average age = 49.34 ± 16.05 years) with lesions in the right hemisphere. All patients were investigated at least 10 months after brain injury. In the group of left-sided brain-lesioned patients, we identified 22 out of the 42 patients in whom touch was impaired for the entire right side of the body. For the group with right-sided lesions, 21 out of the 47 patients presented touch impairments of the entire left side of the body. Thirty-eight out of the 43 patients with hypoesthesia additionally presented mild hemiparesis, whereas in patients without hypoesthesia 20 out of the 27 presented mild hemiparesis. Although the chi-square test did not reveal significant differences between patients with and without hypoesthesia with respect to the presence of paresis (p = 0.12), we additionally accounted for potentially confounding influences by a covariate added to both VLSM models (see “*Lesion mapping”* in Methods). Age, as another potentially confounding factor, was included as a covariate as well.

**Figure 1:**
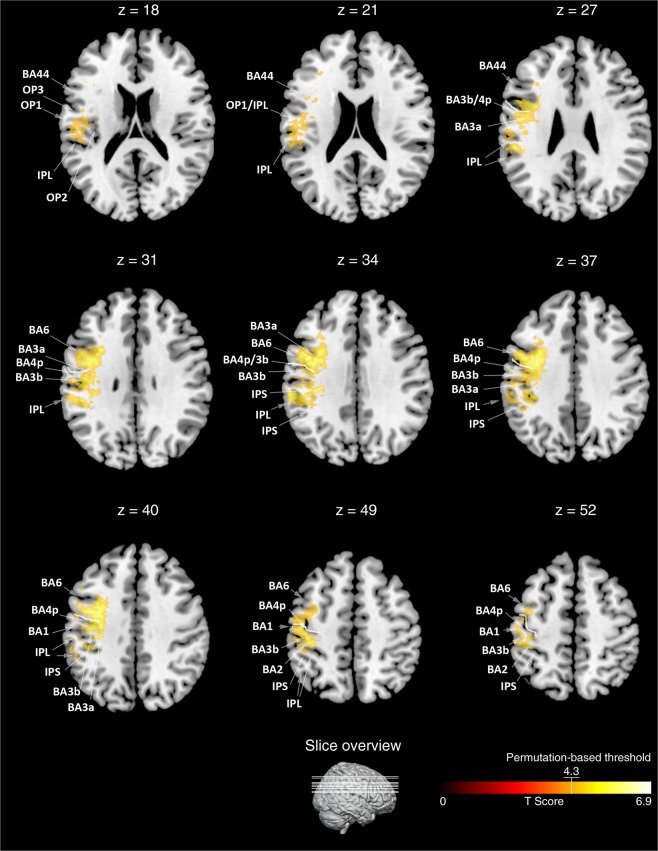
Brain regions associated with hypoesthesia. Results of the VLSM 2.55 t-statistics for the group of left plus “flipped” right brain-lesioned patients. Shown are axial brain slices indexing T-values of the t-statistics (see colour bar at the bottom, orange to yellow). T-statistics comparing hypoesthesia to no hypoesthesia revealed the primary somatosensory areas BA 1, 2, 3a and 3b, BA 4p on the primary motor cortex, the inferior parietal lobe (IPL, BA 40) and intraparietal sulcus (IPS), as well as BA 6 plus BA 44, together assumed to represent the ventral premotor cortex. Downstream to S1 we identified the parietal opercular subdivisions OP 1 to 4, assumed to represent the secondary somatosensory cortex, together with the insular cortex. Numbers above each axial brain slice indicate corresponding z coordinate in MNI space. The white line in brain slices from z = 27 to z = 52 indexes the central sulcus. Colour bar indicates T-scores across lesion maps and white lines crossing the little brain in the last row index the position of the shown brain slices.

**Figure 2:**
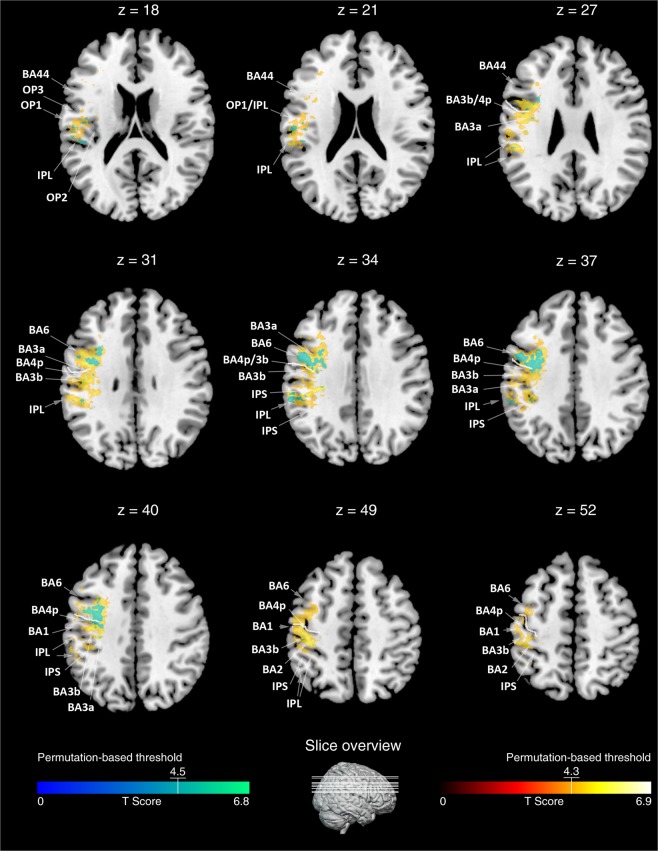
Brain regions associated with hypoesthesia, excluding spatial neglect. Results of the VLSM 2.55 t-statistics excluding patients with right-sided brain lesions and spatial neglect (shown in turquoise). Shown are the same axial brain slices as in Fig. 1. The results of the analysis without neglect patients are superimposed on the statistical map of the whole sample (shown in yellow-orange, same as in Fig. 1). The t-statistics without neglect patients, revealed a smaller topographic extend of hypoesthesia-associated lesions as for the whole group, but nevertheless the same set of brain regions (i.e., primary somatosensory areas BA 1, 2, 3a and 3b, BA 4p on the primary motor cortex, the IPL and IPS, ventral premotor cortex BA 6 plus BA 44, S2-associated parietal opercular subdivisions OP 1 to 4, insular cortex). Numbers above each brain slice indicates corresponding z coordinate in MNI space. The white line in brain slices from z = 27 to z = 52 indexes the central sulcus. Colour bars indicates T-scores across lesion maps (blue to green for lesion maps excluding neglect, red to yellow for the whole sample). White lines crossing the little brain in the last row index the position of the presented brain slices.

**Figure 3:**
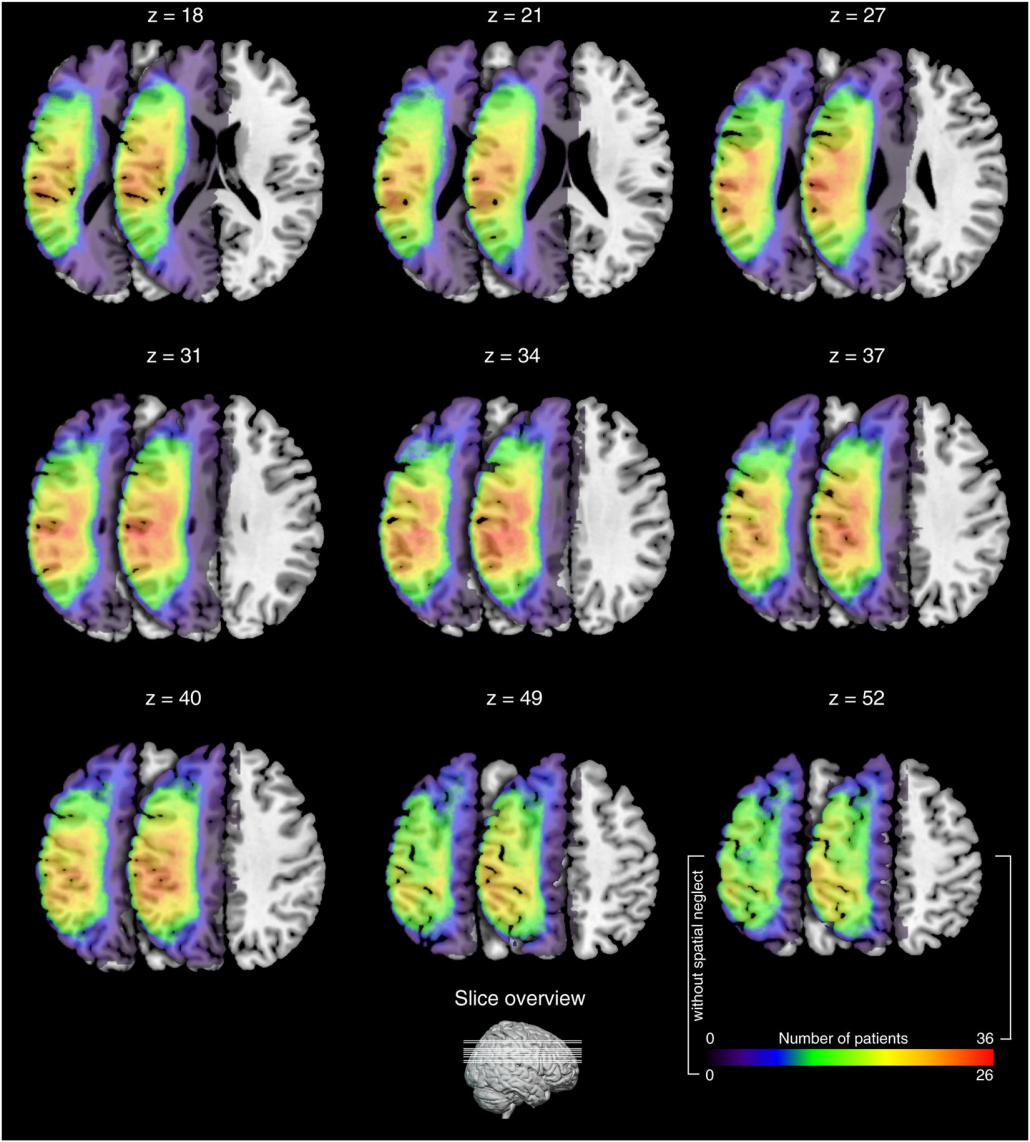
Shown are pairs of the summed lesion maps across patients with and without spatial neglect, superimposed on axial structural T1-weighted MRI images. Left-sided brain slices present the pattern of patients without spatial neglect and the overlapping right-sided brain slices present the pattern of patients with spatial neglect. The colour code indexes number of lesions per voxel. For individual Brodmann areas, age, gender, origin of lesion, and clinical parameters, please refer to Supplementary Table 1. The z-numbers above each brain slice indicates MNI (Montreal Neurological Institute) coordinates.

The t-statistics applied to the left or right sided lesion maps alone did not reveal any significant results, probably due to the more conservative statistical model we applied in the present (t-test, p < 0.05, corrected by 10.000 permutations, VLSM 2.55^18^) as compared to our previous study (Liebermeister measure, corrected false detection rate (FDR) at p < 0.05)^14^. To increase statistical power in the present study, we next added right-sided lesion maps to the left-sided lesion maps after we flipped left-right orientation. T-statistics revealed significant effects of hypoesthesia that overlapped with lesions in the postcentral gyrus (BA 1, 2, 3a, and 3b). On the primary motor cortex (M1), we identified BA 4p, bordering BA 3b within the central sulcus, but not BA4a. More frontally to S1 and BA 4p, we identified BA 6. The effect in BA 6 reached further downwards into the inferior frontal gyrus (BA 44) and was assigned to the ventral premotor cortex. Posterior to the postcentral gyrus, we identified hypoesthesia-associated effects in the neighboured IPL and IPS (Fig. 1). Downstream to S1 and in line with previous studies^14,15^, we identified the parietal opercular subdivisions OP 1 to 4, together with the insular cortex (Fig. 1). Comparable findings were observed for the left or right-sided lesion maps alone, but after lowering the threshold to a non-significant level of p = 0.1. To exclude potentially confounding influences due to spatial neglect, we conducted additional t-tests excluding patients with spatial neglect. Findings convincingly overlapped with the lesion maps obtained for the whole sample (Fig. 2). Figure 3 shows comparable group lesion maps for patients with and without spatial neglect.

## Discussion

Here, we aimed at deciphering those regions in and adjacent to S1 associated with an impaired perception of touch (i.e., hypoesthesia). In S1, hypoesthesia was associated with lesions in each of the four BAs (i.e., BA 1, 2, 3a, 3b)^11–13^. However, not all patients with lesions in BA 3b, as the assumed main entry site of thalamic projections (i.e., SI proper^10^), also presented hypoesthesia. This suggests that thalamic projections do not exclusively target 3b^19^, but probably also neighbouring BAs, such as BA 1, 2, or 3a.

At the anterior border of BA 3b to the primary motor cortex (M1), we identified BA 4p. BA 4p was first described in 1996. Before, M1 has been considered as one structurally and functionally homogeneous area, namely BA 4, occupying the entire precentral gyrus. Geyer *et al*.^20^ were the first who found that BA 4 in the human brain divides into two cytoarchitectonically distinct areas, ‘4 anterior’ (4a) and ‘4 posterior’ (4p). They also showed that roughness discrimination activated BA 4p significantly more than self-generated movements suggesting closer associations to neighbouring S1 (i.e., BA 3b) than to BA4a^20^.

More frontally to BA 4, we identified lesions in the ventral premotor cortex (BA 6, BA 44) associated with hypoesthesia. Electrophysiological recordings from ventral premotor cortex in primates yielded crossmodal receptive fields consisting of multiple body maps^21–24^ as required for multisensory integration processes^24,25^. Also, in humans multi-pattern classification fMRI identified ventral premotor cortex as a multisensory integration region involved in merging crossmodal sensory information from different body maps into a coherent body representation^26^. Damage to these receptive fields may impair the integration of touch perception into a multisensory concept of the body which clinically may manifest as hypoesthesia.

At the posterior border to the postcentral gyrus, we observed significant effects that spread from S1 into sub-areas of the IPL, namely BA 40, and the IPS. This is in line with findings showing IPL activity in response to many different tactile functions, such as spatial discrimination^27^, geometrical shape matching^28^, and detection of deviant tactile stimulations^29^. Based on these and other reports, the IPL was assigned a role in the calibration of attention required for the perception of provoked or spontaneous tactile sensations^16^. The neighbouring IPS seems to represent a key area of the dorsal frontoparietal network involved in shifting the attentional focus and calibration attentional weights^17,30–32^. Attention selection and calibration are mandatory prerequisites for accurate evaluation and classification of sensory inputs, which may explain why we found lesions in both, the IPS and the IPL in association with hypoesthesia.

Downstream to S1, we replicated previously reported associations to hypoesthesia in the parietal operculum, consisting of OP 1 to 4 (S2) and the insular cortex^3,14,15^. As compared to S1, S2 in the parietal operculum consists of larger receptive fields and responds to ipsilateral, as well as contralateral stimuli with greater stimulus selectivity but reduced specificity^3,33–36^. But S2 does not only contribute to touch perception. Human lesion studies indicate its role also in the perception of bodily and surrounding space, since lesions in the right more posteriorly located OP1 and OP2, next to the superior temporal gyrus and ventral postcentral gyrus, cause spatial neglect^37^. To account for potentially confounding influence on group statistics, we additionally analysed lesion maps after excluding patients with spatial neglect. This analysis quarried a VLSM pattern with a smaller topographic extend of hypoesthesia-associated lesions, due to the smaller sample-size, but nevertheless was comparable to the whole-group pattern. This supports the assumption that spatial neglect had no relevant influences on our VLSM analysis.

The insular cortex, as the assumed interface for bottom-up cognitive signals, seems to underpin affective processing and awareness of sensory signals^38,39^, hence contributing to the sense of ownership and agency^40^. The insula is divided by the central sulcus into an anterior and a posterior part. While the posterior part seems to subserve bodily awareness^41^, the anterior part appears to process viscerosensory^42^ and interoceptive signals^43^. Our findings generally support these assumptions and agree with functional brain imaging studies yielding insula activity in response to nocuous events^44^, vibrations^45,46^, affective touches^47^, and non-painful mechanical stimuli^48^.

## Conclusions

In line with our a-priori hypotheses, we identified regions, together assumed to constitute a path of perception and recognition that leads through S1 and S2, in the parietal operculum, to the insula^3,14,15^. Posteriorily to S1, we found the IPL and IPS, as expected; regions implemented in attention selection and calibration processes^16,17^. In our previous lesion-mapping study^14^, we focussed on brain regions downstream to S1 and additionally identified the putamen as a subcortical structure involved in the perception of touch. Since our group statistics in the present study did not reveal the putamen, we investigated the corresponding area voxel by voxel. We found that most patients with lesions in the putamen (mean across the putamen: n = 10) did not present hypoesthesia, whereas only few patients (mean across the putamen: n = 2) presented hypoesthesia. This finding is not in line with our previous study. Together with the rather low number of lesioned voxels per se, the discrepancies between both our studies question whether lesions in the putamen contribute to hypoesthesia.

Besides expected effects in S1, S2, the insular cortex^3,14,15^, the IPL and the IPS^16,17^, present lesion-mapping additionally quarried BA 4p - a primary motor area involved in motor control but which also proved to be sensitive to tactile stimulations^20^. More ventrally, we moreover identified the ventral premotor cortex; a brain region assumed to contain neuronal ensembles involved in whole-body perception^26^. To rule out, that these effect in motor cortices were caused by differences in the presence of hemiparesis, we compared hemiparesis between patients with and without hypoesthesia and revealed no significant differences. We also analysed lesion maps without accounting for the presence of paresis which resembled the same hypoesthesia-associated brain pattern as shown in this article (not shown to avoid redundancies). This supports the interpretation that hypoesthesia-associated effects in motor (BA 4p) and premotor cortex (BA 6/44) were not just driven by additional motor impairments but by hypoesthesia. We also addressed potentially confounding influences by spatial neglect by two t-tests, one including patients with neglect (Fig. 1), and the other one excluding patients with neglect (Fig. 2). Maps of both t-tests convincingly overlapped. To exclude age as another confounding factor, we added age as a covariate. Resulting brain maps, however, showed an almost similar pattern as the maps revealed without regressing out age.

Together, our findings are in agreement with intracerebral recordings in humans showing that more than 10% of the cortical surface appears to be involved in the processing of somatosensory stimuli. The identified cortical network convincingly overlaps with the network presented in the present study, including not only S1, S2 and insular areas, but also neighbouring motor, premotor, and inferior parietal areas^49^. These and the present findings emphasize the role of those regions in the perception of touch. Whether the impairment in touch perception results from an impairment in perceptual awareness, attention or multisensory integration processes required for proper body perception remains speculative. With respect to the existing body of evidence^26^, we assume that lesions in the ventral premotor cortex impair multisensory integration processes required for the correct integration of tactile stimuli into a whole-body concept required for correct perceptual awareness. Lesions in the IPL and the IPS may in turn impair correct attentional selection and calibration processes^16,17^, required to accurately evaluate the intensity and location of touches applied to the skin. The loss in associated capacities may trigger or facilitate the impression of hypesthesia when being touched. Lesion studies with isolated lesions in each of those regions would be required to further disentangle their true functional anatomical associations, but suitable patients are rare and currently available statistical methods are unsuitable to handle small samples or single cases.

## Methods

### Patients

We included patients with brain lesions in the postcentral gyrus extending into the parietal, temporal and/or pre-/frontal lobe in the same brain hemisphere. Patients with malformations or lesions in the contralateral brain hemisphere were excluded. Cognitive and severe communication deficits were exclusion criteria too. The study was approved by the ethics committee of the University Clinic Leipzig and conducted according to the ethical guidelines of the Declaration of Helsinki. All patients gave written informed consent.

Besides a high-resolution whole-brain 3D standard T1-weighted anatomical image, we used a T2-weighted fluid-attenuated inversion-recovery (FLAIR) image in parallel to better delineate the borders of stroke lesions. Data acquisition was done with a 3 T Bruker MedSpec 100 System (Bruker, Ettlingen, Germany), a 3 T Tim TRIO, or a 3 T VERIO MRI scanner (Siemens, Erlangen, Germany).

### Examination of touch impairments (i.e., hypoesthesia)

The examination procedures were the same as described elsewhere^14^. The standardized examinations were conducted by physiotherapists of the MPI Clinic for Cognitive Neurology. Patients lay supine on an examination bed. They were told to close their eyes and the room was darkened. A paintbrush (6 mm tip width) was used to apply strokes to the fingers or toes. From there, strokes were directed further proximally to the upper limb and from there to the trunk and the face. With each stroke, the patient was asked whether the sensation was felt as normal or attenuated (i.e., hypoesthesia). Distal and proximal strokes were compared, as well as the left and the right side of the body. To identify the extension of hypoesthesia, strokes were next directed from the body part with hypoesthesia to neighbouring body parts using a paintbrush with a 14 mm tip-width. This was repeated on the dorsal body site.

### Lesion mapping

We used MRIcroN (http://www.sph.sc.edu/comd/rorden/mricron) to manually delineate the lesion on every single transversal T1- weighted image slice. The two researchers (S.P. and B.P.) who created the 3D lesion maps were unaware of the clinical deficits. They first delineated the lesions independently of each other. Afterwards, both examiners reviewed all maps together. In case of discrepancies between both examiners, the lesion map was jointly revised. Lesion maps together with the T1-weighted image were spatially normalized to the MNI-template (i.e, Montreal Neurological Institute) using SPM12 (http://www.fil.ion.ucl.ac.uk/spm/).

To assess the relationship between impaired touch perception (i.e., hypoesthesia) and brain lesions, normalized lesion maps were applied to the VLSM software 2.55 (https://langneurosci.mc.vanderbilt.edu/resources.html) for Matlab R2017b (Natick, Massachusetts, USA, https://mathworks.com) that divides patients according to whether they present a lesion in a given voxel or not. These two groups are then statistically compared (i.e., t-test) according to whether they present a symptom, like hypoesthesia^18^. The analysis is corrected for the lesion extend by regressing out the number of lesioned voxels.

To assess the influence of neglect on group results, we conducted two analyses: The first analysis included all patients with lesions in the left hemisphere. In patients with lesions in the right hemisphere, we first flipped right/left orientation before adding the lesion masks. The second analysis was conducted in the same way after excluding patients with spatial neglect. In both analyses, presence of paresis (either present or not) was added as a covariate to account for the corresponding variance. Voxels surviving the alpha level of p < 0.05 and 10.000 permutations were considered significant.

To identify lesion-associated Brodmann areas in each patient, we superimposed them to the Brodmann template offered by MRIcroN (Supplementary Table 1). The assignment of the group findings to their corresponding BAs was done by superimposing the group maps on the cytoarchitectonic maps as implemented in the Anatomy toolbox for SPM^50^ (Fig. 1). Insula cortex, BA 6 and BA 44 were allocated using the WFU_PickAtlas by Joseph Maldjian from the Functional MRI Laboratory at the Wake Forest School of Medicine (see http://www.nitrc.org/projects/wfu_pickatlas).

## Supplementary information

Supplementary Table 1
