# Supplementary material for: Prefrontal and posterior parietal contributions to the perceptual awareness of touch
Source: Sci Rep. 2019 Nov 18;9:16981. doi: 10.1038/s41598-019-53637-w (PMC6861260; doi:10.1038/s41598-019-53637-w)
Supplement: Supplementary file 1 — Supplementary Table 1 [file 41598_2019_53637_MOESM1_ESM.docx]

Supplementary Information

Prefrontal and posterior parietal contributions

to the perceptual awareness of touch

by Rullmann M. ^1,2^, Preusser S.^1^, Pleger B.^1,3,4^

^1^ Department of Neurology, Max Planck Institute for Human Cognitive and Brain Sciences, Stephanstr. 1a, 04103 Leipzig, Germany

^2^ Department of Nuclear Medicine, University Hospital Leipzig, Liebigstr. 20, 04103 Leipzig, Germany

^3^ Department of Neurology, BG University Hospital Bergmannsheil, Ruhr-University Bochum, Bürkle-de-la-Camp Plz. 1, 44789 Bochum, Germany

^4^ Collaborative Research Centre 874 Integration and Representation of Sensory Processes, Ruhr-University Bochum, Universitätsstr. 150,44801 Bochum, Germany

Supplementary Table 1. Patients’ clinical and demographical parameters. The table lists patients’ age, gender, handedness, origin of lesion, lesioned BAs, deficits in touch perception, presence of paresis and spatial neglect.

| **Patient** | **Age (years)** | **Gender** | **Handedness** | **Origin of Lesion** | **Hemisphere of Brain Damage** | **Location of Brain Damage (Brodmann Areal)** | | | | **Hypoesthesia** | **Paresis** | **Neglect** |
| --- | --- | --- | --- | --- | --- | --- | --- | --- | --- | --- | --- | --- |
|  |  |  |  |  |  | **frontal** | **parietal** | **temporal** | **occipital** | **0 = no 1 = yes** | **0 = no 1 = yes** | **0 = no 1 = yes** |
| P1 | 43 | FeMale | Right | Partial MCA Stroke | Left | 04 05 06 07 08 09 10 11 44 45 46 47 | 01 02 03 39 40 43 | 21 22 41 42 | - | 1 | 1 | 1 |
| P2 | 74 | Male | Right | Partial MCA Stroke | Right | 4 | 01 02 03 | - | - | 1 | 1 | 0 |
| P3 | 51 | FeMale | Right | Partial MCA Stroke | Left | - | 01 02 03 40 | 20 21 | - | 0 | 1 | 0 |
| P4 | 33 | Male | Right | Partial MCA Stroke | Right | 04 05 06 07 09 44 47 | 01 02 03 39 40 43 | 21 22 41 42 52 | - | 1 | 1 | 0 |
| P5 | 29 | Male | Right | Partial MCA Stroke | Left | 04 06 09 44 45 | 01 02 03 07 39 40 43 | 21 22 41 42 | - | 1 | 1 | 0 |
| P6 | 49 | Male | Right | Partial MCA Stroke | Left | 04 11 12 | 01 02 03 39 40 | 21 | - | 1 | 1 | 0 |
| P7 | 60 | Male | Right | Partial MCA Stroke | Right | 04 06 38 44 45 | 02 05 07 40 43 | 21 22 38 41 42 | 19 | 1 | 1 | 1 |
| P8 | 38 | Male | Right | Partial MCA Stroke | Right | 04 06 08 09 11 12 44 45 46 | 01 02 03 05 07 39 40 43 | 21 22 38 41 42 52 | - | 1 | 0 | 0 |
| P9 | 53 | Male | Right | Partial MCA Stroke | Left | 04 06 09 44 45 46 47 | 01 02 03 05 07 39 40 43 | 21 22 | - | 1 | 1 | 1 |
| P10 | 37 | FeMale | Right | Partial MCA Stroke | Right | 04 06 40 | 01 02 03 05 07 | 20 21 38 41 42 | - | 1 | 1 | 0 |
| P11 | 59 | Male | Right | Partial MCA Stroke | Right | 29 31 32 33 | 01 02 03 05 07 23 | - | - | 0 | 1 | 0 |
| P12 | 73 | FeMale | Right | Partial MCA Stroke | Right | 04 06 08 09 44 45 46 | 01 02 03 05 40 43 | 41 42 | - | 1 | 0 | 1 |
| P13 | 62 | Male | Right | Partial MCA Stroke | Right | 47 | 02 05 07 39 | - | - | 0 | 1 | 0 |
| P14 | 62 | Male | Right | Partial MCA Stroke | Right | 04 06 43 44 45 47 | 01 02 03 39 40 | 21 22 37 38 41 42 52 | - | 0 | 1 | 1 |
| P15 | 71 | Male | Right | Partial MCA Stroke | Right | 4 | 03 07 | - | 17 18 19 | 0 | 1 | 0 |
| P16 | 50 | Male | Right | Partial MCA Stroke; Partial ACA Stroke | Right | 04 08 09 46 | 01 02 03 05 07 | - | 17 18 19 | 1 | 1 | 1 |
| P17 | 24 | Male | Right | Traumatic brain injury | Left | 08 09 10 11 44 45 46 47 | 01 02 03 | - | - | 0 | 0 | 0 |
| P18 | 55 | FeMale | Right | Partial MCA Stroke | Right | 04 06 08 09 | 01 02 03 05 39 40 | 20 21 22 38 41 42 52 | 17 18 19 | 1 | 1 | 1 |
| P19 | 64 | Male | Right | Partial MCA Stroke; Partial ACA Stroke | Right | 04 08 09 23 24 33 46 | 01 02 03 05 07 31 39 40 | - | 19 | 0 | 0 | 1 |
| P20 | 23 | Male | Right | Traumatic brain injury; Partial MCA Stroke | Right | 04 10 11 12 44 45 46 47 | 01 02 03 05 07 43 | 20 21 22 38 41 42 52 | - | 1 | 1 | 1 |
| P21 | 60 | Male | Right | Traumatic brain injury | Left | 09 44 46 | 01 02 03 43 | 22 41 | - | 1 | 0 | 0 |
| P22 | 51 | FeMale | Right | Partial MCA Stroke | Left | 04 06 08 09 46 | 01 02 03 40 43 | 21 22 38 | - | 1 | 1 | 1 |
| P23 | 52 | FeMale | Right | Partial MCA Stroke | Left | 6 | 02 03 40 | - | - | 0 | 1 | 0 |
| P24 | 38 | FeMale | Right | Partial MCA Stroke | Left | 22 | 01 02 03 40 | 41 52 | - | 1 | 1 | 0 |
| P25 | 20 | Male | Right | Traumatic brain injury | Right | 04 06 08 09 10 11 12 24 25 32 | 01 02 03 05 07 | - | - | 0 | 1 | 0 |
| P26 | 35 | Male | Right | Traumatic brain injury | Left | 04 06 08 44 | 01 02 03 05 40 43 | 20 28 34 38 | - | 1 | 1 | 0 |
| P27 | 66 | Male | Right | Partial MCA Stroke; Partial ACA Stroke; Partial PCA Stroke | Right | 04 06 08 09 10 11 | 01 02 03 05 07 39 40 | 20 21 37 | 18 19 | 1 | 0 | 1 |
| P28 | 56 | Male | Right | Partial MCA Stroke | Right | 4 | 01 02 03 05 07 39 40 | 22 41 42 | - | 0 | 1 | 0 |
| P29 | 56 | Male | Right | Partial MCA Stroke | Right | 04 06 08 09 44 45 46 47 | 01 02 03 05 07 40 43 | - | - | 1 | 1 | 0 |
| P30 | 26 | Male | Right | Traumatic brain injury; Partial MCA Stroke | Right | 04 06 08 09 44 45 46 47 | 01 02 03 05 07 39 40 43 | 20 21 22 37 38 41 42 | - | 1 | 1 | 1 |
| P31 | 54 | FeMale | Right | Traumatic brain injury; Partial MCA Stroke | Right | 04 06 19 39 | 01 02 03 07 39 40 | - | 19 | 1 | 1 | 1 |
| P32 | 36 | Male | Right | Partial MCA Stroke; Partial PCA Stroke; Subarachnoid hemorrhage | Right | 06 10 11 | 02 05 39 40 | - | 18 19 | 1 | 1 | 0 |
| P33 | 35 | Male | Right | Traumatic brain injury; Tumor | Left | 04 06 08 09 24 32 44 45 46 47 | 01 03 | 37 38 | - | 0 | 0 | 0 |
| P34 | 40 | Male | Right | Partial MCA Stroke; Subarachnoid hemorrhage | Right | 04 08 09 | 01 02 03 | - | - | 0 | 0 | 0 |
| P35 | 65 | Male | Left | Partial MCA Stroke | Right | 4 | 03 07 | - | - | 0 | 1 | 0 |
| P36 | 21 | Male | Right | Traumatic brain injury | Right | 04 06 08 09 10 11 44 45 46 47 | 01 02 03 05 07 39 40 43 | 21 22 37 38 41 42 | - | 1 | 1 | 1 |
| P37 | 39 | Male | Right | Traumatic brain injury | Left | 04 06 08 09 | 01 02 03 | - | - | 0 | 1 | 0 |
| P38 | 49 | FeMale | Right | Partial MCA Stroke | Left | 04 06 | 01 02 03 | - | - | 0 | 1 | 0 |
| P39 | 16 | Male | Right | Tumor | Left | - | 02 40 | - | - | 0 | 0 | 0 |
| P40 | 60 | Male | Right | Partial MCA Stroke; Partial ACA Stroke | Left | 04 08 | 01 02 03 | - | - | 0 | 1 | 0 |
| P41 | 60 | Male | Right | Partial MCA Stroke | Right | 04 06 08 44 47 | 01 02 03 40 43 | 20 21 22 38 41 | - | 1 | 1 | 1 |
| P42 | 50 | FeMale | Right | Partial MCA Stroke; Partial ACA Stroke | Right | 04 06 08 09 10 11 24 32 33 44 45 46 47 | 01 02 03 05 07 31 39 40 43 | 21 22 37 38 41 42 | 18 19 | 1 | 1 | 1 |
| P43 | 61 | FeMale | Right | Partial MCA Stroke | Left | 04 06 08 09 11 44 45 46 47 | 01 02 03 40 43 | 41 42 | 22 | 1 | 1 | 1 |
| P44 | 32 | Male | Right | Partial MCA Stroke; Subarachnoid hemorrhage | Left | 04 06 08 09 10 11 12 23 24 25 26 28 29 30 32 33 34 44 45 46 47 | 01 02 03 05 07 31 39 40 43 | 20 21 22 28 35 36 37 38 41 42 48 52 | - | 1 | 1 | 0 |
| P45 | 59 | Male | N/A | Partial MCA Stroke | Right | 04 06 08 09 10 11 44 45 46 47 | 01 02 03 05 39 40 43 | 21 22 38 41 42 | - | 1 | 1 | 1 |
| P46 | 47 | Male | Right | Partial MCA Stroke | Left | 04 06 44 45 47 | 01 02 03 40 43 | 22 | - | 0 | 1 | 0 |
| P47 | 52 | Male | Right | Partial MCA Stroke | Left | 04 06 | 01 02 03 07 39 40 | - | 18 19 | 1 | 1 | 0 |
| P48 | 52 | Male | Right | Partial MCA Stroke; Partial ACA Stroke | Left | 04 06 08 09 10 11 12 24 25 32 33 44 45 46 47 | 01 02 03 05 43 | - | - | 1 | 1 | 0 |
| P49 | 46 | Male | Right | Partial MCA Stroke | Left | 04 06 08 09 24 | 01 02 03 05 07 31 40 | - | - | 1 | 1 | 0 |
| P50 | 48 | FeMale | Right | Partial MCA Stroke | Left | 04 06 11 44 45 46 47 | 01 02 03 39 40 43 | 20 21 22 28 34 35 36 37 38 41 42 | 19 | 0 | 1 | 0 |
| P51 | 60 | Male | Right | Partial MCA Stroke | Left | 04 06 08 09 10 11 44 45 46 47 | 01 03 43 | - | - | 0 | 1 | 0 |
| P52 | 44 | FeMale | Right | Partial MCA Stroke | Left | 04 06 08 09 39 40 44 45 46 47 | 01 02 03 40 | 21 22 37 | - | 1 | 1 | 0 |
| P53 | 57 | Male | Right | Partial MCA Stroke | Right | 04 06 08 09 10 11 41 42 44 45 46 47 | 01 02 03 39 40 43 | 20 21 22 28 35 36 37 38 | 19 | 1 | 1 | 1 |
| P54 | 62 | FeMale | Right | Partial MCA Stroke | Left | 04 06 44 45 46 47 | 01 02 03 39 40 43 | - | - | 1 | 1 | 0 |
| P55 | 41 | Male | N/A | Encephalitis | Right | - | 02 05 07 39 | 20 21 37 38 | 19 | 0 | 0 | 0 |
| P56 | 61 | Male | Left | Partial MCA Stroke | Left | 04 06 09 10 11 44 45 46 47 | 01 02 03 39 40 43 | 21 22 37 41 42 | - | 1 | 1 | 0 |
| P57 | 28 | Male | Right | Partial MCA Stroke | Right | 04 06 45 | 03 05 07 39 | 20 21 37 | 18 19 | 0 | 1 | 1 |
| P58 | 59 | FeMale | Right | Partial MCA Stroke | Right | 04 06 | 01 02 03 40 43 | - | - | 1 | 1 | 0 |
| P59 | 24 | Male | N/A | Partial MCA Stroke | Left | - | 01 02 05 39 40 43 | 21 22 37 38 41 42 | - | 1 | 0 | 0 |
| P60 | 22 | Male | Right | Traumatic brain injury | Right | 4 | 01 02 03 05 | 20 21 22 35 36 38 | - | 0 | 1 | 0 |
| P61 | 51 | FeMale | N/A | Tumor | Left | 04 06 08 09 20 21 22 37 39 40 41 42 43 44 46 | 01 02 03 05 07 | - | - | 1 | 1 | 0 |
| P62 | 59 | Male | N/A | Traumatic brain injury | Left | 04 06 08 09 10 11 12 24 32 44 45 46 47 | 01 02 03 23 43 | 20 21 22 28 34 35 36 38 41 42 | - | 0 | 1 | 1 |
| P63 | 56 | Male | N/A | Partial MCA Stroke | Left | 04 06 08 09 44 45 46 47 | 01 02 03 39 40 43 | - | - | 1 | 1 | 0 |
| P64 | 67 | FeMale | Right | Partial MCA Stroke | Left | 04 06 | 01 02 03 05 07 39 40 43 | 22 41 42 | - | 1 | 1 | 0 |
| P65 | 37 | FeMale | N/A | Partial MCA Stroke | Right | 04 06 11 44 45 47 | 01 02 03 05 39 40 43 | 21 22 37 38 41 42 | 19 | 1 | 1 | 0 |
| P66 | 49 | FeMale | Right | Partial ACA Stroke; Subarachnoid hemorrhage | Left | 04 08 09 10 24 32 | 01 02 03 05 07 23 31 | - | - | 0 | 1 | 0 |
| P67 | 64 | Male | N/A | Partial MCA Stroke | Right | - | 02 05 07 39 | - | 19 | 0 | 0 | 1 |
| P68 | 49 | Male | N/A | Partial MCA Stroke | Right | 04 06 44 45 47 | 01 02 03 39 40 43 | 22 38 41 42 | - | 0 | 1 | 1 |
| P69 | 42 | Male | N/A | Partial MCA Stroke | Left | 04 06 44 45 47 | 01 02 03 39 40 43 | 22 38 41 42 | - | 1 | 1 | 0 |
| P70 | 42 | Male | N/A | Partial MCA Stroke | Left | 04 06 08 09 10 11 44 45 46 47 | 01 02 03 05 07 40 43 | 20 21 22 38 41 42 | - | 1 | 1 | 0 |
